# Load Balancing in Cloud Computing Environment Using Improved Weighted Round Robin Algorithm for Nonpreemptive Dependent Tasks

**DOI:** 10.1155/2016/3896065

**Published:** 2016-02-03

**Authors:** D. Chitra Devi, V. Rhymend Uthariaraj

**Affiliations:** Ramanujam Computing Centre, Anna University, Chennai 600 025, India

## Abstract

Cloud computing uses the concepts of scheduling and load balancing to migrate tasks to underutilized VMs for effectively sharing the resources. The scheduling of the nonpreemptive tasks in the cloud computing environment is an irrecoverable restraint and hence it has to be assigned to the most appropriate VMs at the initial placement itself. Practically, the arrived jobs consist of multiple interdependent tasks and they may execute the independent tasks in multiple VMs or in the same VM's multiple cores. Also, the jobs arrive during the run time of the server in varying random intervals under various load conditions. The participating heterogeneous resources are managed by allocating the tasks to appropriate resources by static or dynamic scheduling to make the cloud computing more efficient and thus it improves the user satisfaction. Objective of this work is to introduce and evaluate the proposed scheduling and load balancing algorithm by considering the capabilities of each virtual machine (VM), the task length of each requested job, and the interdependency of multiple tasks. Performance of the proposed algorithm is studied by comparing with the existing methods.

## 1. Introduction

Cloud computing is a computing paradigm for managing and delivering services over the internet and is defined as “a model for enabling ubiquitous, convenient, on-demand network access to a shared pool of configurable computing resources (e.g., networks, servers, storage, applications, and services) that can be rapidly provisioned and released with minimal management effort or service provider interaction” [1]. Cloud computing is an integrated concept of parallel and distributed computing which shares resources like hardware, software, and information to computers or other devices on demand. With the aid of cloud computing and internet facility, the customer can access the aforementioned resources by paying for the duration of use. Virtual machine (VM) is an execution unit that acts as a foundation for cloud computing technology. Virtualization consists of creation, execution, and management of a hosting environment for various applications and resources. The VMs in the cloud computing environment share resources like processing cores, system bus, and so forth. The computing resources available for each VM are constrained by total processing power. In this model of environment the job arrival pattern is unpredictable and also the capabilities of each virtual machine vary from one another. Hence, load balancing becomes a critical task leading to a poor system performance and maintaining stability. Thus, it becomes imperative to develop an algorithm which can improve the system performance by balancing the work load among virtual machines. There are various load balancing algorithms available, such as round robin, weighted round robin, dynamic load balancing, Equally Spread Current Execution (ESCE) Algorithm, First Come First Serve, Ant Colony algorithm, and Throttled algorithm. The most frequently used scheduling techniques for a nonpreemptive system are first in first out (FIFO) and weighted round robin (WRR) [2]. CloudSim-3.0.3 is the simulation environment for the cloud computing research works. It supports both system and behaviour modelling of cloud system components such as data centres, hosts, virtual machines (VMs), and resource provisioning policies. It supports modelling and simulation of cloud computing environments consisting of both single and internetworked clouds (federation of clouds). It exposes custom interfaces for implementing scheduling and load balancing policies of jobs into VMs and provisioning techniques for allocation of VMs under internetworked cloud computing scenarios. It can leverage virtualized services even on the fly based on requirements (workload patterns and QoS) varying with time. In the present work, the custom-built static, dynamic scheduling, and custom-built load balancing are implemented as an improved weighted round robin (IWRR) to achieve the higher performance and utilization of VMs under varying load patterns. This intern produced the comparatively faster response time to the client's request on the application jobs.

*(1) Objective*. The Cloud computing has to assign the computational tasks to the most suitable virtual machines from the dynamic pool of the VMs by considering the requirements of each task and the load of the VMs. The requests from the clients are directed to any of the data centers in the cloud. Then again the same requests are directed by the data center to the most suitable VMs based on the cloud management policies depending on the load of the individual VMs. The two most frequently used scheduling principles in a nonpreemptive system are round robin and the weighted round robin (WRR) policies. The round robin policy does not consider the resource capabilities, priority, and length of the tasks. So, the higher priority and lengthy tasks end up with the higher response times. The weighted round robin considers the resource capabilities of the VMs and assigns higher number of tasks to the higher capacity VMs based on the weightage given to each of the VMs. But it failed to consider the length of the tasks to select the appropriate VM, whereas the proposed and implemented algorithm (improved WRR algorithm) additionally considers the length and priority of the tasks in addition and selects the appropriate VM to execute the tasks for the lower response times.

The objective is to optimize the performance of virtual machines using the combination of static and dynamic load balancing by identifying the length of the jobs, resource capabilities, interdependency of multiple tasks, effectively predicting the underutilized VMs, and avoiding the overload on any of the VMs. This additional parameter of “job length” consideration can help schedule the jobs into the right VMs at any moment and is able to deliver the response in a very minimum execution time. The effective scheduling on this algorithm will also minimize the overload on a VM and subsequently it will also minimize the task migrations.

The performance of the improved WRR algorithm was analyzed and evaluations of the algorithm with respect to the existing round robin and weighted round robin algorithm were carried out. This work considers that the job contains multiple tasks and the tasks have interdependency between them. A job can use multiple VMs for its various tasks to complete its entire processing instruction. Also, the task can use the multiple processing elements of a single VM based on the configuration and availability.

*(2) Organization of the Work*. The paper is organized as follows.  Section 2 discusses related works.  Section 3 discusses scheduling and load balancing with design and algorithms.  Section 4 provides model for our design.  Section 5 provides experimental results and performance analysis.  Section 6 concludes our research work and points out future work.

## 2. Related Works

Load balancing of nonpreemptive dependent tasks on virtual machines (VMs) is an important characteristic of task scheduling in clouds. Whenever certain VMs are overloaded, the load has to be shared with the under loaded VMs to achieve the optimal resource utilization for the least completion time of tasks. Moreover, the overhead impact on identifying the resource utilization and task migration has to be considered in the load balancing algorithms. Broadly, the VM uses two different task execution mechanisms like space shared and time shared. In space shared mechanism the tasks will be executed one after the other. It implies that only one task per CPU/core is executed in its CPU. The remaining tasks assigned to that VM should be in the waiting queue. As a result, the task migration in the load balancing will be easier on this space shared mechanism by identifying a task in the waiting queue of the overloaded VM and assigning it to the underloaded VM. Nevertheless, in time shared mechanism, the tasks are executed concurrently in a time sliced manner which resembles the execution of tasks in parallel mode. Here, the task migration in the load balancing will be highly complicated due to the time sliced execution of all the tasks. So, almost 90% of the time, a certain percentage amount of instructions of the tasks will be in the completed state on the time sliced mechanism. The decision of identifying the task to be migrated from the higher loaded VM to lower loaded VM is very expensive due to the loss of the previously completed portion in the higher loaded VM and the job's earlier execution impact on the other jobs execution time in the higher loaded VM. So, scheduling and load balancing algorithm should attain the optimal/minimal migration of tasks with equal load distribution between the resources based on its resource capability without any idle time of any of the resources at any point of time in the overall combined resource execution time. This method/process helps attain the optimal/minimal execution time in the cloud environment. Algorithm should also consider the unpredictable nature of job arrivals and its allocation to the suitable VMs by considering the jobs with multitasks and its interdependencies between them. Algorithm should be suitable for both homogenous and heterogeneous environments on varied job lengths. With this objective in mind the literatures has been analyzed and the proposal of the algorithm has been reached.

In [2], the Honey Bee Behavior inspired load balancing algorithm was proposed, which aims to achieve well balanced load across VMs to maximize the throughput and to balance the priorities of tasks on the VMs. Hence, the amount of waiting time of the tasks in the queue is minimal. Using this algorithm average execution time and reduction in waiting time of tasks on queue were improved. This algorithm works for heterogeneous type of systems and for balancing nonpreemptive independent tasks.

In [3], the importance and significance of performance optimization and power reduction in data centers for cloud computing and queuing model for a group of heterogeneous multicore servers with different sizes and speeds were discussed. In particular, it addresses the problem of optimal power allocation and load distribution for multiple heterogeneous multicore server processors across clouds and data centers. Nevertheless, it is only a feasibility study for modelling power.

The elasticity of cloud infrastructures enables a suitable platform for execution of deadline-constrained workflow applications [4]. To mitigate effects of performance variation of resources on soft deadlines of workflow applications, an algorithm that uses idle time of provisioned resources and budget surplus to replicate tasks is proposed. This reduces the total execution time of applications as the budget available for replication increases. Static job arrival is also modelled, whereas overhead involvement of duplicate executions and run time arrival of jobs is not taken into account.

“Skewness” [1] is the metric to measure the unevenness of a server with multidimensional resource utilization. By minimizing skewness, the different types of workloads have been combined to improve the overall utilization of server resources. The significant contributions of this work are that they developed a resource allocation system that can avoid overload in the system effectively while minimizing the number of servers used. They designed a load prediction algorithm that can capture the future resource usages of applications accurately without looking inside the VMs. The algorithm can capture the rising trend of resource usage patterns and help reduce the placement churn significantly. QoS parameters such as response time or completion time of tasks are not discussed.

In [5], an enhanced scheduling in weighted round robin for the cloud infrastructure services was proposed, which considers job length and resource capabilities. This type of algorithm minimizes the response time of the jobs by optimally utilizing the participating VMs using static and dynamic scheduling by identifying the length of the jobs and resource capabilities and effectively predicting the underutilized VMs and avoiding the overload on any of the VMs. The multilevel interdependent tasks have been considered. Load balancing in the heavily loaded scenarios for the task migrations has not been considered.

In [6–8] scheduling algorithm for dependent task in grid was proposed. Efficient mapping of the DAG based application was proposed by [9, 10]. This algorithm is based on the list scheduling approach. A noncritical path earliest-finish scheduling algorithm for heterogeneous computing was proposed by [11]. This algorithm shows that a higher performance can be achieved by applying to the heterogeneous computing environment. Similar type of problem was discussed in [12]. Stochastic hill climbing approach was used for load distribution in cloud computing [13], in which the soft computing based approach has been compared with round robin and First Come First Serve.

A dynamic workflow scheduling technique for grid and cloud computing environment [14] minimizes the workflow execution time and reduces the scheduling overhead. Scheduling of scientific workflows using genetic algorithm called Chaos Genetic algorithm was discussed in [15] to solve the scheduling problem considering both user's budget and deadline. This algorithm produces better results within a shorter time. Similar type of problems was discussed by [16–19].

In [20] job scheduling algorithm in cloud environment was discussed by considering priority of jobs as a main QoS parameter. Moreover, this algorithm considers three important issues like complexity, consistency, and makespan. Keeping the objective in mind literature has been analyzed and the proposal of the algorithm has been reached.

## 3. Scheduling and Load Balancing

**Figure 1:**
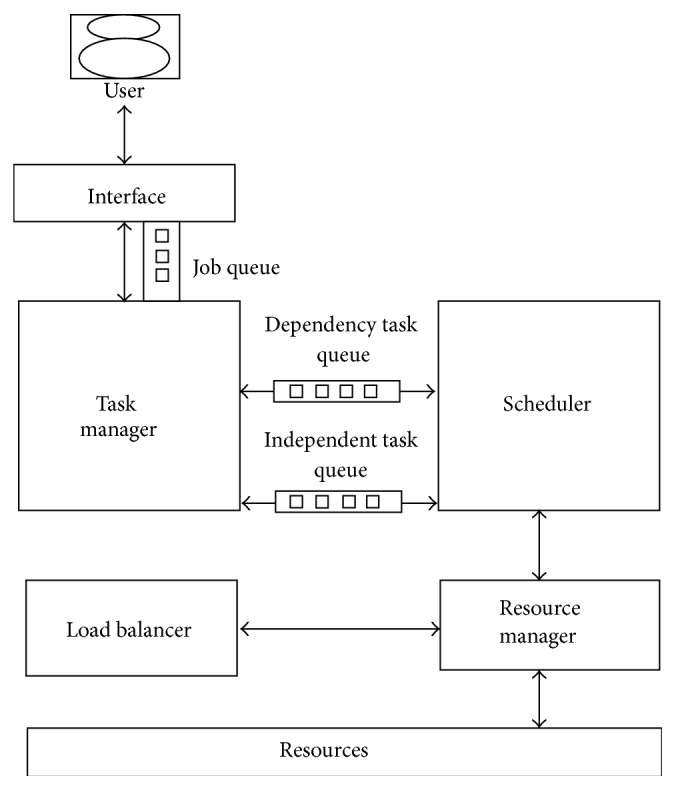
Scheduling and load balancing design.

Figure 1 shows the scheduling and load balancing design, in which the scheduler has the logic to find the most suitable VM and assign the tasks to VMs based on the proposed algorithm. The scheduler places the run time arrival jobs in the most suitable VMs based on the least utilized VM at that particular job arrival time.

Load Balancer decides the migration of task from a heavily loaded VM to an idle VM or least loaded VM at run time, whenever it finds an idle VM or least loaded VM by utilizing the resources current status information. Resource monitor communicates with all the VMs resource prober and collects the VM capabilities, current load on each VM, and number of jobs in execution/waiting queue in each VM. The task requirement is provided by the user which includes the length of the tasks to be executed and transfers the requirements to the scheduler for its operative decisions.

### 3.1. Scheduling and Load Balancing Design

Job request is given by the user through the interface and passed to task manager for dependency and independent task analysis. This module receives the job and verifies whether the job is a complete independent task or contains multiple tasks. If it contains multiple tasks, then it verifies the interdependency between the multiple tasks. The dependency task queue and independent task queue are found. The dependent tasks will be notified to the scheduler so that parent tasks are scheduled after child tasks are executed. Dependency task queue will contain the tasks, which depends on the other tasks present in the VMs. Once all the child tasks present in this queue completed its execution the parent task will be taken for the execution by assigning it to the VM, whereas independent task queue contains independent tasks. Independent task queue and dependency task are input to the scheduler. The scheduler selects the appropriate VM based on IWRR algorithm. This scheduler collects the resources information from the resource manager. It calculates the processing capacity of each of the VMs and then it applies the proposed algorithm to find the appropriate VM for the given job. Additionally, every VM is maintaining the JobExecutionList, JobPauseList, and JobWaitingList information specific to it. The JobExecutionList contains the current executing job list and the JobPausedList contains the temporarily paused jobs in the VM. Similarly the JobWaitingList Queue contains the waiting jobs on the specific VM, but this will be executed upon receiving the JobExecutionList, JobPauseList, and JobWaitingList from each of the VMs; calculation of the least utilized VM is carried out for every request arrival. Then, this least utilized VM information will be returned to the scheduler. Resource manager communicates with all the VMs to collect each of its capabilities by getting its number of the processing elements and its processing capacity to each of its elements. This resource manager additionally calculates the weightage to each of the VMs based on the processing capacity allotted to it. This also identifies the memory configured available in each of the VMs. Load balancer calculates the ratio between the number of jobs running and the number of VMs. If the ratio is less than 1, then it communicates the scheduler to identify a VM for the job; else it will calculate the load on each of the VMs using the job execution list of the VMs. If the utilization is less than the 20%, then the least utilized VM will be allotted; else the scheduler will be communicated to identify the most suitable VM for the job. Once the appropriate VM has been identified, the Job will be assigned to that VM. The configured data centers include hosts and their VM with corresponding processing elements form the set of resources available for computing. The resources are probed for idleness and for heavy load so that the job requests are effectively allocated to an appropriate resource.

### 3.2. Computation of Load Imbalance Factor

The sum of loads of all virtual machines is defined as

(1) $$\begin{array}{c} L=\overset{k}{\underset{i=1}{\sum }}l_{i}, \end{array}$$

where *i* represents the number of VMs in a data center.

The load per unit capacity is defined as

(2) LPC=L∑i=1mciThreshold  Ti=LPC∗ci,

where *c*_*i*_ is the capacity of the node.

The load imbalance factor of a particular virtual machine is given by

(3) $$\begin{array}{c} If\;\;VM\left\{\begin{array}{cc} <\left|T_{i}-\overset{k}{\underset{v=1}{\sum }}L_{v}\right|, & Underloaded,\\ >\left|T_{i}-\overset{k}{\underset{v=1}{\sum }}L_{v}\right|, & Overloaded,\\ =\left|T_{i}-\overset{k}{\underset{i=1}{\sum }}l_{i}\right|, & Balanced. \end{array}\right. \end{array}$$

The migration of task from the overloaded VM to underloaded VM can be allowed until the load on the overloaded VM drops below the threshold and the difference is *μ*_*i*_.

A virtual machine is identified as underloaded where the sum of loads of all the VMs is below the threshold value of that VM. The underloaded VM accepts the load from the overloaded VM until the load on the VM exceeds the threshold and the difference is *λ*_*j*_ as shown below.

The transfer of load from the overloaded VM is carried out until its load is less than the threshold. The underloaded VM can accept load only up to its threshold, thus avoiding it being overloaded. This implies that the amount of load that can be transferred from the underloaded VM should be in the range of *μ* and *λ*.

### 3.3. Algorithms

The two most frequently used scheduling principles in a nonpreemptive system are round Robin and weighted round robin policies. Improved weighted round robin is the proposed algorithm. Existing algorithms are implemented for comparative analysis.

#### 3.3.1. Round Robin Algorithm

The round robin algorithm allocates task to the next VM in the queue irrespective of the load on that VM. The Round Robin policy does not consider the resource capabilities, priority, and the length of the tasks. So, the higher priority and the lengthy tasks end up with the higher response times.

#### 3.3.2. Weighted Round Robin Algorithm

The weighted round robin considers the resource capabilities of the VMs and assigns higher number of tasks to the higher capacity VMs based on the weightage given to each of the VMs. But it failed to consider the length of the tasks to select the appropriate VM.

#### 3.3.3. Improved Weighted Round Robin Algorithm

The proposed improved weighted round robin algorithm is the most optimal algorithm and it allocates the jobs to the most suitable VMs based on the VM's information like its processing capacity, load on the VMs, and length of the arrived tasks with its priority. The static scheduling of this algorithm uses the processing capacity of the VMs, the number of incoming tasks, and the length of each task to decide the allocation on the appropriate VM.

The dynamic scheduling (at run time) of this algorithm additionally uses the load on each of the VMs along with the information mentioned above to decide the allocation of the task to the appropriate VM. There is a probability at run time that, in some of the cases, the task may take longer execution time than the initial calculation due to the execution of more number of cycles (like a loop) on the same instructions based on the complicated run time data.

In such situations, the load balancer rescues the scheduling controller and rearranges the jobs according to the idle slot available in the other unutilized/underutilized VMs by moving a waiting job from the heavily loaded VMs. The load balancer identifies the unutilized/underutilized VMs through resource prober whenever a task is completed in any of the VMs. If there is no unutilized VM, then the load balancer will not take up any task migration among the VMs. If it finds any unutilized/underutilized VM, then it will migrate the task from the overloaded VM to the unutilized/underutilized VM. The load balancer analyses the resource's (VM) load only on the completion of any of the tasks on any of the VMs. It never examines the resource's (VM) load independently at any time to circumvent the overhead on the VMs. This will help in reducing the number of task migrations between the VMs and the number of resource probe executions in the VMs.

### 3.4. Implementation Aspect of the Algorithm

The system implementation consists of five major modules:

- Static scheduler (initial placements).
- Dynamic scheduler (run time placements).
- Load balancer (decision on job migration at run time).
- Task interdependent scheduler.
- Resource monitor.

The static scheduler has the function to find the most suitable VM and assign the tasks to VMs based on the algorithms (simple round robin, weighted round robin, and improved weighted round robin) applied in the scheduler. The dynamic scheduler has the function to place the run time arrival jobs to the most suitable VMs based on the least utilized VM at that particular job arrival time. Load balancer/scheduler controller decides the migration of task from a heavily loaded VM to an idle VM or least loaded VM at run time whenever it finds an idle VM or least loaded VM by utilizing the resource monitor information. Resource monitor communicates with all the VMs resource probers and collects the VM capabilities, current load on each VM, and number of jobs in execution/waiting queues in each VM to decide the appropriate VMs to the jobs. The task requirement estimator identifies the length of the tasks to be executed and transfers the estimated results to the load balancer for its operative decisions.

**Figure 2:**
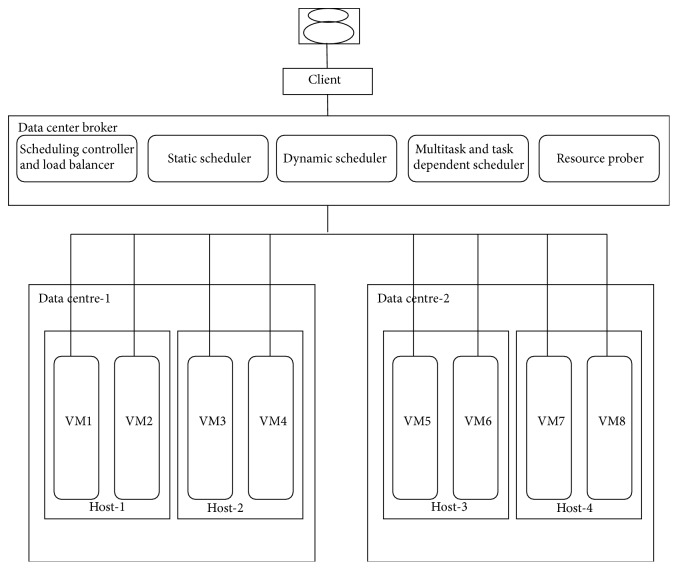
System architecture.

Figure 2 illustrates the system architecture, in which the system consists of the data center broker and multiple data centers. The data center can host any number of hosts and this intern can host any number of VMs in each of the hosts based on its capacity. The data center broker has the important components to schedule and load-balance the jobs effectively, whereas the algorithms of these components vary according to the round robin, weighted round robin, and the improved weighted round robin load balancing.

#### 3.4.1. Dynamic Scheduler in IWRR Load Balancer

Dynamic scheduling in IWRR load balancer is achieved by Initialization, mapping (scheduling), load balance, and execution as explained in Algorithm 1. Initialization is done by collecting the pending MI execution time from each of the created VMs and arranging it in ascending order of pending time followed by arranging the run time of the arrived tasks in queue, based on the priority. Mapping (scheduling) involves selection of task which is in top of the queue and calculation of its completion time in each VM. Then task is assigned to the most appropriate VM based on completion and pending execution time. Load balancing is done by adding the corresponding tasks and execution time to the VMs pending time. Then rearrange the VM utilized list based on the latest load, which is followed by sending the task to VM processing queue and adding the task to the assigned list. When all cloudlets are assigned to VMs, the tasks are executed in assigned VMs.

#### 3.4.2. Load Balancer in IWRR

Load balancer initializes by collecting the pending execution time from each of the created VMs then arranging it in ascending order to identify the number of tasks in each VM and arrange it in increasing order queue. Mapping (scheduling) is done by getting a task from VM with higher pending time and then identifies the most suitable VM to execute this task by calculating the completion time of this task in all the VMs and assigning the selected task to the identified VM. Finally, load balancing is accomplished by rearranging the order, based on the new execution time in each VM (refer to Algorithm 2).

#### 3.4.3. Task Dependency Queue in Multilevel Interdependent Tasks

**Figure 3:**
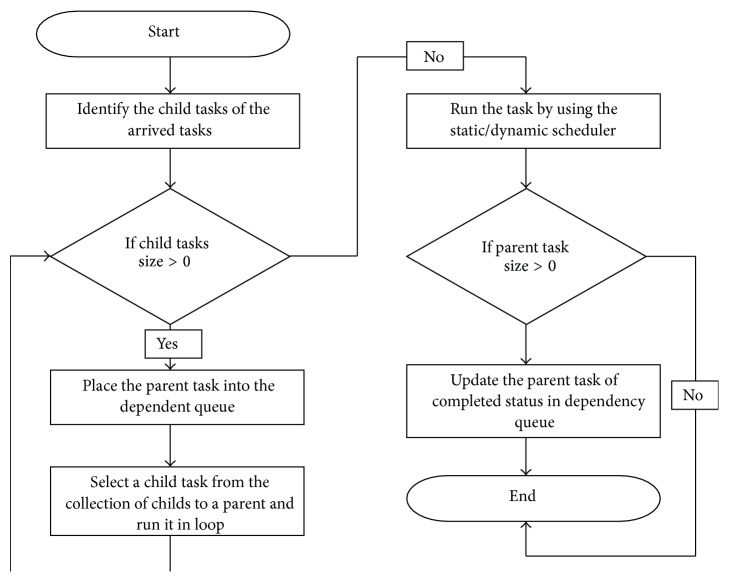
Flow chart of multilevel interdependency tasks.

Multilevel interdependency tasks are explained in Figure 3.

## 4. Model

Let VM = (VM_1_, VM_2_,…, VM_*m*_) be the set “*m*” of virtual machines, which should process “*n*” tasks represented by the set *T* = (*T*_1_, *T*_2_,…, *T*_*n*_). All the VMs are running in parallel and are unrelated and each VM runs on its own resources. There is no sharing of its own resources by other VMs. We schedule nonpreemptive dependent tasks to these VMs, in which “*n*” tasks assigned to “*m*” VMs are represented as an LP model from (4) to (5).

*Processing Time.* Let PT_*ij*_ be the processing time of assigning task “*i*” to VM “*j*” and define

(4) $$\begin{array}{c} x_{ij}=\left\{\begin{array}{cc} 1, & \text{if task “}i\text{” is assigned},\\ 0, & \text{otherwise}. \end{array}\right. \end{array}$$

Then the linear programming model is given as

(5) Minimize Z=∑i=1n∑j=1mPTijxijSubject  to: ∑i=1nxij=1,j=1,2,…,m xij=0 or 1.

*Resource Utilization*. Maximizing the resource utilization is another important objective which is derived from (6) and (7). Achieving high resource utilization becomes a challenge. Now average utilization is defined as [21]

(6) $$\begin{array}{c} Average\;\;utilization=\frac{\sum_{j\in VMs}^{}{CT}_{j}}{Makespan*Number\;\;of\;\;VMs}, \end{array}$$

where makespan can be expressed as

(7) $$\begin{array}{c} Makespan=max\left\{\;\frac{{CT}_{j}}{j\in VMs}\;\right\}. \end{array}$$

*Capacity of a VM.* Consider

(8) $$\begin{array}{c} C_{VM}={pe}_{num}*{pe}_{mips}, \end{array}$$

where *C*_VM_ is the capacity of the VM (see (8)), pe_num_ is the number of processing elements in the VM, and pe_mips_ is the million instructions per second of a PE.

*Capacity of All the VMs.* Consider

(9) $$\begin{array}{c} C=\overset{m}{\underset{j=1}{\sum }}C_{{VM}_{j}}, \end{array}$$

where *C* is the summation of capacities of all VMs, the capacity allotted to the application/environment (refer to (9)).

*Task Length.* Consider

(10) $$\begin{array}{c} TL=T_{mips}*T_{pe}. \end{array}$$

*Job Length.* Consider

(11) $$\begin{array}{c} JL=\overset{p}{\underset{k=1}{\sum }}{TL}_{i}, \end{array}$$

where “*p*” is the number of interdependent tasks for the job.

*Task Load Ratio*. Task load ratio is calculated in (12) and (13) to identify and allocate the tasks to virtual machines. It is defined as

(12) TLRij=TLiCVMj,i=1,2,…,n  tasks,  j=1,2,…,m  Virtual  machines,

where TL_*i*_ is the task length which is estimated at the beginning of the execution and *C*_VM_ is the capacity of the VM. Consider

(13) $$\begin{array}{c} If\;\;{TLR}_{ij}=\left\{\begin{array}{cc} 0, & \text{assign the task to VM},\\ Otherwise, & \text{Do not assign}. \end{array}\right. \end{array}$$

## 5. Experiment Results and Performance Analysis

The performance of the IWRR algorithm has been analyzed based on the results of simulation done in the CloudSim. The classes of the CloudSim simulator have been extended (overridden) to utilize the newly written algorithm. In the following illustrations, the response time, number of job migrations, cumulative idle time of all tasks, and the number of delayed tasks are analyzed in the RR, WRR, and IWRR algorithms under the combination of heterogeneous and homogenous job lengths with heterogeneous resource conditions. Configuration details are given in Table 1.

### 5.1. Homogeneous Tasks on Heterogeneous Resources (VMs)

#### 5.1.1. Comparison of Overall Execution Time

*Analysis*. The following is the order of highest to lowest performance of the algorithms in the provided homogenous jobs on heterogeneous environment:

(a) improved weighted round robin with job length, (b) weighted round robin, and (c) round robin.

**Figure 4:**
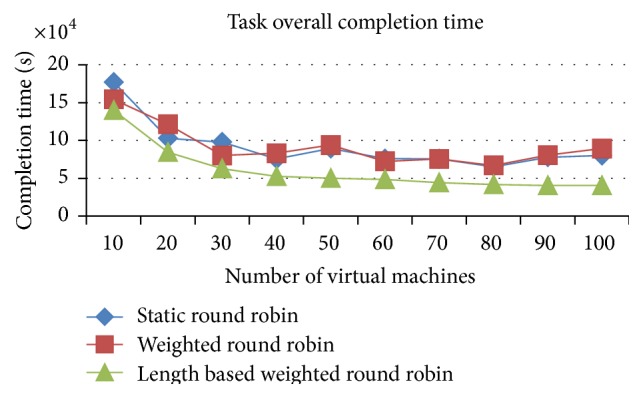
Execution completion time (space shared).

**Figure 5:**
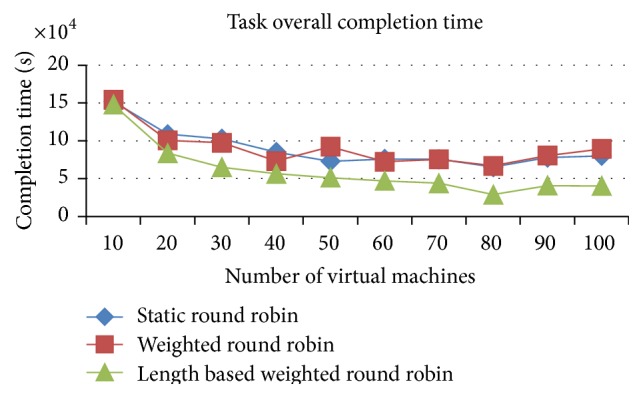
Execution completion time (time shared).

Figures 4 and 5 proved that the IWRR by job length delivers a faster completion time than the other 2 load balancing algorithms (RR and WRR) in the heterogeneous resources (VMs) and homogenous jobs. The IWRR's static scheduler algorithm considers the job length along with processing capacity of the heterogeneous VMs to assign the job. So, more number of jobs gets assigned to the higher capacity VMs in the homogenous jobs on heterogeneous environments. This helps complete the job in a shorter time. The dynamic scheduler considers the load of all its configured VMs and its tentative completion time of the current load has been identified. Then, the scheduler calculates the arrived job's estimated completion time in each of the configured VMs and adds this calculated timing with the existing load's completion time on each VM. Now, the least possible completion time has been identified from the above calculations for this particular job in one of the VMs and then the job has been assigned to this VM. So this algorithm is most suitable to the heterogeneous environment data centers.

The load balancer in the IWRR with job length runs at the end of each task's completion. If the load balancer finds any of the VMs completes all its assigned tasks, then it will identify the highly loaded VM from the group and it calculates the possible completion time of those jobs present in the highly loaded VM and the least loaded/idle VM. If the least loaded VM can finish any of the jobs present in the highly loaded VM in the shortest possible time, then that job will be moved to the least loaded VM.

The WRR considers the ratio of the VM capacity to the total VM capacity and it assigns the proportionate number of arrived jobs into the VM. So it performs in the next level. But if any lengthy jobs are assigned to the low capacity VMs based on the above calculation, then this will delay the execution completion time.

The simple RR has not considered any variables about the environment, VM capabilities, and the job lengths. It simply assigns the jobs to the VM lists one after another in an ordered manner. So its completion time of the jobs is higher than the other 2 algorithms.

#### 5.1.2. Comparison of Task Migration

**Figure 6:**
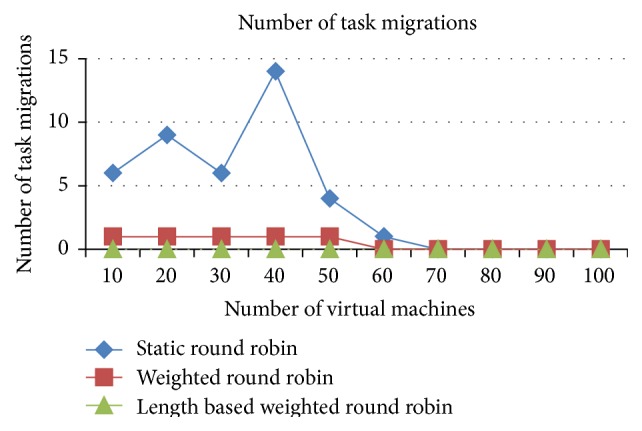
Number of task migrations (space shared).

**Figure 7:**
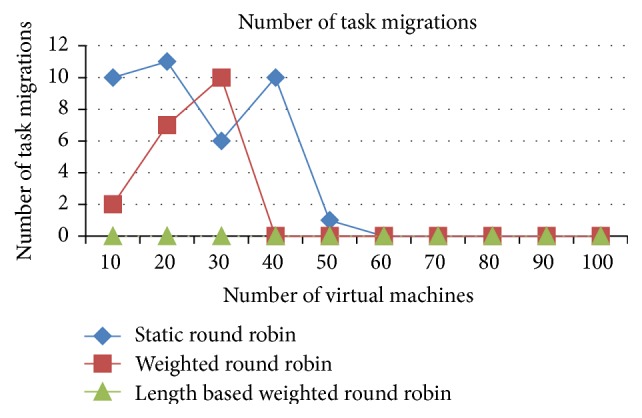
Number of task migrations (time shared).

The task migrations are very minimal in the IWRR algorithm due to extensive static and dynamic scheduler algorithm in identifying the most appropriate VM to each of the jobs which is illustrated in Figures 6 and 7. So, the load balancer has been unable to find the further optimization to complete the task in the shortest time. But in the case of WRR and RR algorithms the static and dynamic scheduler has not considered the job lengths. Instead, it considers only the resource capabilities and the arrived job list. So, the load balancer has been able to find the further optimization at run time, and it shifts the jobs from higher loaded VM to the underutilized VMs. This intern produces the higher task migrations in the weighted round robin and round robin algorithms. The number of task migrations in the lower number of resources is high in the WRR and RR algorithms.

#### 5.1.3. Comparison of Delayed Tasks and Combined Idle Time of All Tasks (Space Shared)

**Figure 8:**
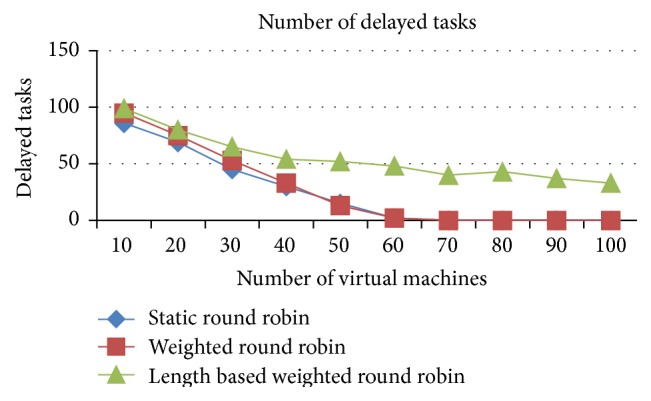
Number of delayed tasks (space shared).

**Figure 9:**
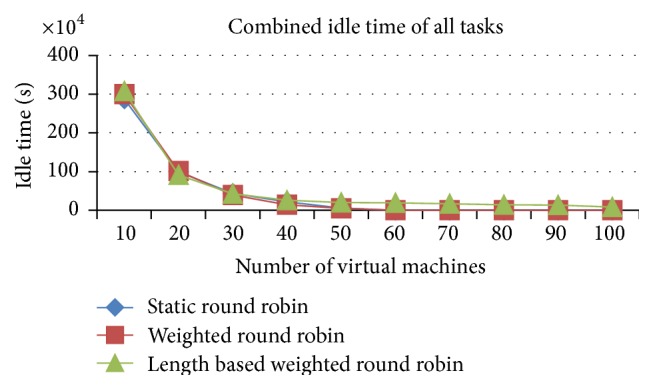
Idle time of all tasks (space shared).

The number of delayed tasks and the combined idle time of all tasks are higher in the IWRR than the other two algorithms, which is analyzed from Figures 8 and 9. This increase happened due to the allocation of more tasks in the higher capacity VMs. At any point of time only one job can run in the space spared CPU/PE, even if it has a higher capacity PE. So, if another job got allocated to the same VM due to its higher processing capacity, then that job has to be in waiting state until the running job gets completed. This increases the number of delayed tasks and the combined idle time in the IWRR algorithm. But in the other two algorithms, the jobs get assigned to even the lower capacity VMs without accurately predicting the possible completion time in various VMs. So, the number of delayed tasks and combined idle time of all tasks are lower in RR and WRR.

#### 5.1.4. Comparison of Million Instructions Reexecuted due to Task Migration

**Figure 10:**
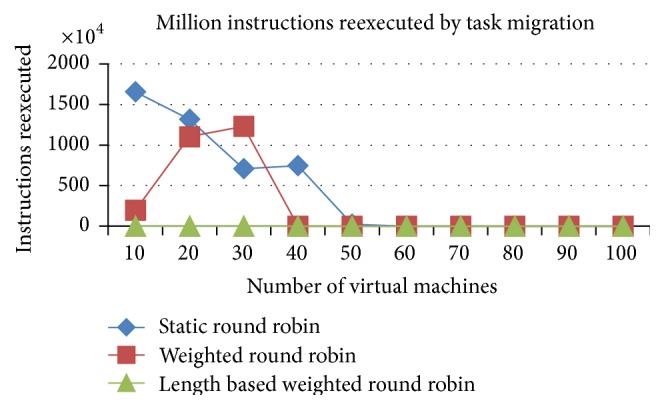
Million instructions reexecuted (time shared).

*Analysis*. The job migrations from one VM to another VM lead to the job's execution termination in one of the VMs. Otherwise, the current state of the execution has to be copied over to another VM to proceed from the left out location in the previous allotted VM. In this work, the current state of the job at the time of job migration will be lost. So, the job will be reexecuted from the start of its instructions in the migrated VM. This kind of execution wastage comes in the time shared CPU rather than the space shared CPU. The instruction reexecution will be higher in the RR and WRR algorithms due to the higher number of task migrations in RR and WRR (refer to Figure 10).

### 5.2. Heterogeneous Tasks on Heterogeneous Resources (VMs)

#### 5.2.1. Comparison of Overall Execution Time

*Analysis*. The following is the order of highest to lowest performance of the algorithms in the provided heterogeneous environment:

(a) improved weighted round robin with job length, (b) weighted round robin, (c) round robin.

**Figure 11:**
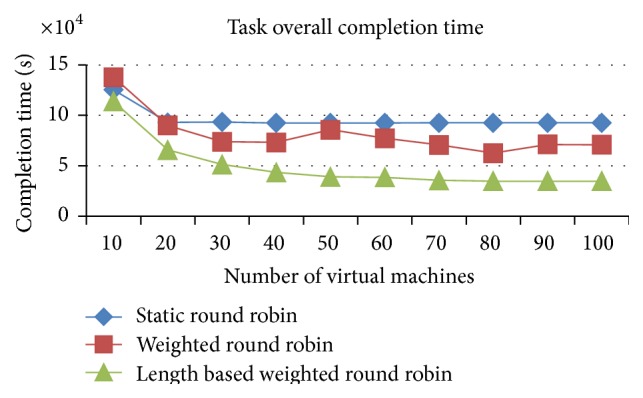
Execution completion time (space shared).

**Figure 12:**
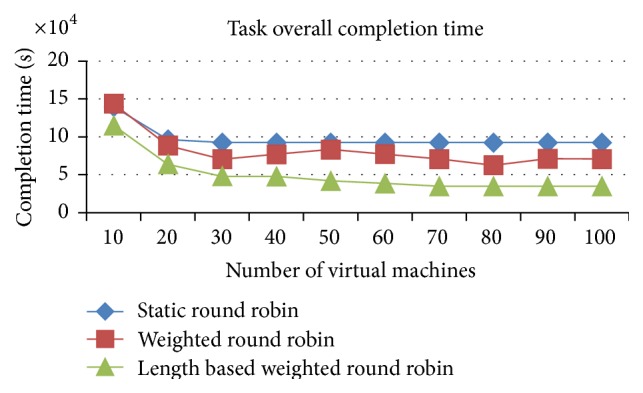
Execution completion time (time shared).

Figures 11 and 12 proved that the improved weighted round robin by job length delivers a faster completion time than the other two load balancing algorithms (RR and WRR) in the heterogeneous resources (VMs) and heterogeneous jobs. The IWRR's static scheduler algorithm considers the job length along with processing capacity of the heterogeneous VMs to assign the job. So, the lengthy jobs get assigned to the higher capacity VMs in the heterogeneous environments. This helps complete the job in a shorter time. The dynamic scheduler considers the load of all its configured VMs and its tentative completion time of the current load has been identified. Then, the scheduler calculates the arrived job's estimated completion time in each of the configured VMs and adds this calculated timing with the existing load's completion time on each VM. Now, the least possible completion time has been identified from the above calculations for this particular job in one of the VMs and then the job has been assigned to this VM. So this algorithm is most suitable to the heterogeneous environment data centers.

The load balancer in the IWRR with job length runs at the end of each task's completion. If the load balancer finds any of the VMs completes all its assigned tasks, then it will identify the highly loaded VM from the group and it calculates the possible completion time of those jobs present in the highly loaded VM and the least loaded/idle VM. If the least loaded VM can finish any of the jobs present in the highly loaded VM in the shortest possible time, then that job will be moved to the least loaded VM. The WRR considers the ratio of the VM capacity to the total VM capacity and it assigns the proportionate number of arrived jobs into the VM. So it performs in the next level. But if any lengthy jobs are assigned to the low capacity VMs based on the above calculation, then this will delay the execution completion time. The simple RR has not considered any variables about the environment, VM capabilities, and the job lengths. It simply assigns the jobs to the VM lists one after another in an ordered manner. So its completion time of the jobs is higher than the other two algorithms.

#### 5.2.2. Comparison of Task Migration

**Figure 13:**
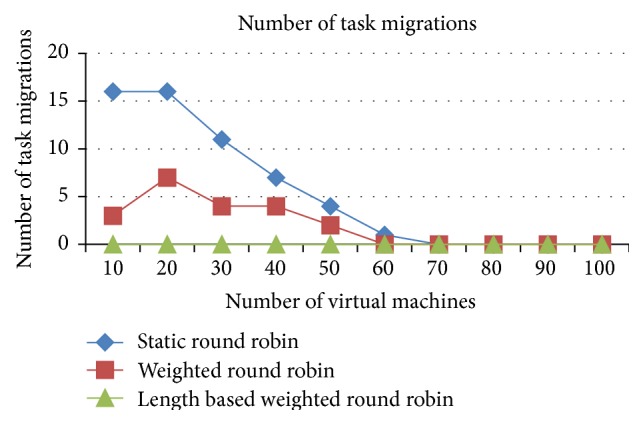
Number of task migrations (space shared).

**Figure 14:**
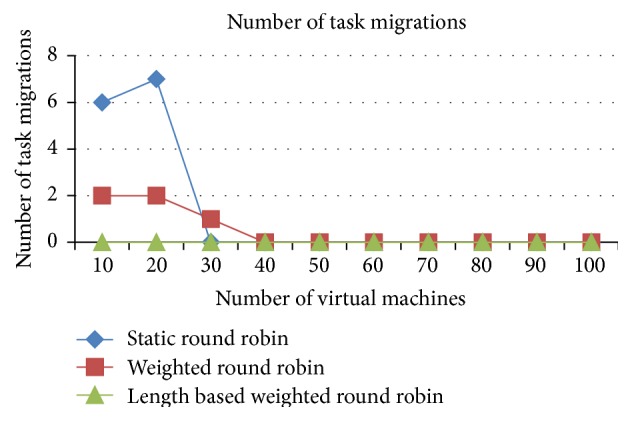
Number of task migrations (time shared).

*Analysis*. The task migrations are very minimal in the IWRR algorithm due to extensive static and dynamic scheduler algorithm in identifying the most appropriate VM to each of the jobs which is derived from Figures 13 and 14. So, the load balancer has been unable to find the further optimization to complete the task in the shortest time. But in the case of WRR and RR algorithms the static and dynamic scheduler has not considered the job lengths. Instead, it considers only the resource capabilities and the arrived job list. So, the load balancer has been able to find the further optimization at run time, and it shifts the jobs from higher loaded VM to the underutilized VMs. This intern produces the higher task migrations in the WRR and RR algorithms. Additionally these numbers of task migrations are high in the lower number of resources in the WRR and RR algorithms.

#### 5.2.3. Comparison of Delayed Tasks and Combined Idle Time of All Tasks

**Figure 15:**
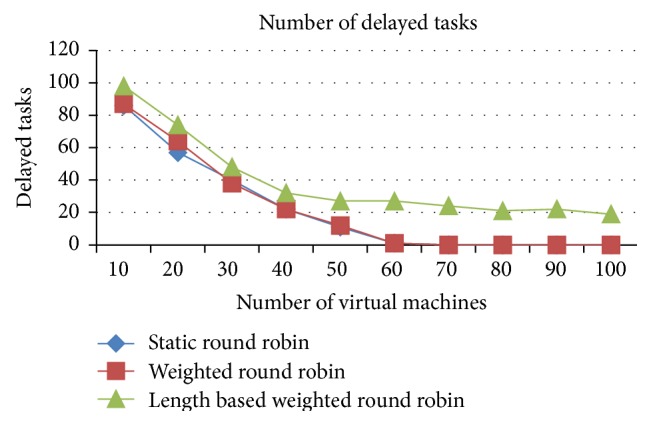
Number of delayed tasks (space shared).

**Figure 16:**
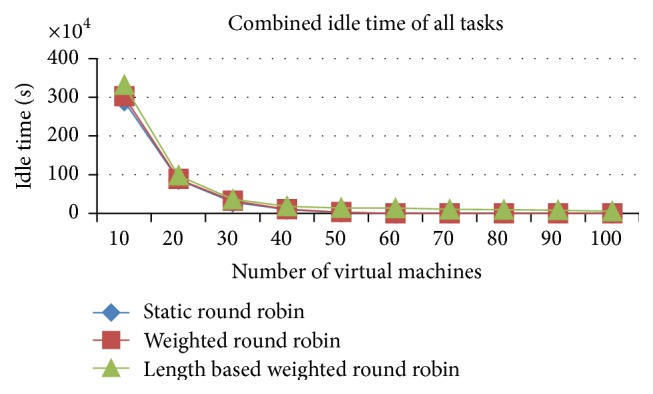
Idle time of all tasks (space shared).

*Analysis*. Figures 15 and 16 explain that the number of delayed tasks and the combined idle time of all tasks are higher in the IWRR than the other two algorithms. This increase happened due to the allocation of more tasks in the higher capacity VMs. At any point of time only one job can run in the space spared CPU/PE, even if it has a higher capacity PE. So, if another job got allocated to the same VM due to its higher processing capacity, then that job has to be in waiting state until the running job gets completed. This increases the number of delayed tasks and the combined idle time in the IWRR algorithm. But in the other two algorithms, the jobs get assigned even to the lower capacity VMs without accurately predicting the possible completion time in various VMs. So, the number of delayed tasks and combined idle time of all tasks are lower in RR and WRR.

#### 5.2.4. Comparison of Million Instructions Reexecuted due to Task Migration

**Figure 17:**
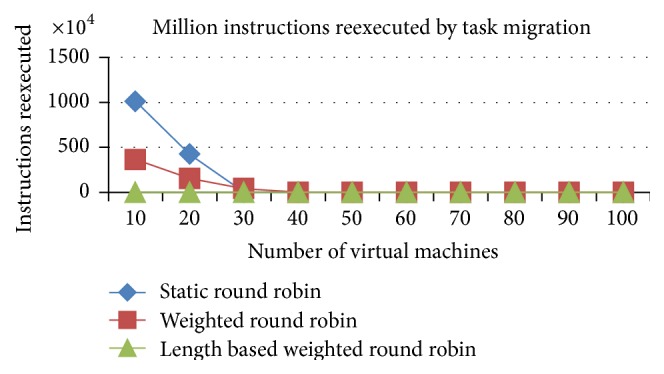
Million instructions reexecuted (time shared).

*Analysis*. The job migrations from one VM to another VM lead to the job's execution termination in one of the VMs. Otherwise, the current state of the execution has to be copied over to another VM to proceed from the left out location in the previous allotted VM. In this project, the current state of the job at the time of job migration will be lost. So, the job will be reexecuted from the start of its instructions in the migrated VM. This kind of execution wastage comes in the time shared CPU. The instruction reexecution will be higher in the RR and WRR algorithms due to the higher number of task migrations in RR and WRR, which is shown in Figure 17.

## 6. Conclusion and Future Enhancements

In this work, the improved weighted round robin algorithm considers the capabilities of each VM and the task length of each requested job to assign the jobs into the most appropriate VMs. This improved weighted round robin algorithms are having three different stages to handle the three different scenarios in the environment life cycle. The static scheduler algorithm pays attention to the initial placement of the jobs, which distributes the job requests to the participating VMs evenly based on the VM's capabilities and the length of the requested job. The dynamic scheduler considers the load of all its configured VMs and its tentative completion time of the current load has been identified along with the arrived job's estimated completion time in each of the configured VMs. After this, the least possible completion time has been identified from the above calculations for this particular job in one of the VMs and then the job has been assigned to this VM. The load balancer in the improved weighted round robin runs at the end of each task's completion. This always makes the loads evenly distributed across all the VMs at the end of each task's completion and thus eliminates any idle time in the participating resources (VMs).

The performance analysis and experiment results of this algorithm proved that the improved weighted round robin algorithm is most suitable to the heterogeneous/homogenous jobs with heterogeneous resources (VMs) compared to the other round robin and weighted round robin algorithms. This algorithm considers the response time as the main QoS parameter.

As part of the future enhancements, we can consider multiple PEs in the participating heterogeneous VMs along with the heterogeneous multiple PEs capable jobs with distributed computing capabilities in the improved weighted round robin algorithm. Additionally, the load balancing can also consider transferring the state of jobs between the VMs in the job migrations. These above considerations can help in further reducing the job completion time in all the algorithms.

This work had considered the overall completion time of all the participating jobs in different algorithms. Instead, in the future enhancements, the completion time of each job can be compared in the different scheduling and load balancing algorithms. The algorithms can be fine-tuned further to achieve the better consistent results on all the different perspectives. Similarly, the comparison results should be taken for the different job arrival patterns on all the three different scheduling and load balancing algorithms.

## Figures and Tables

**Algorithm 1:**
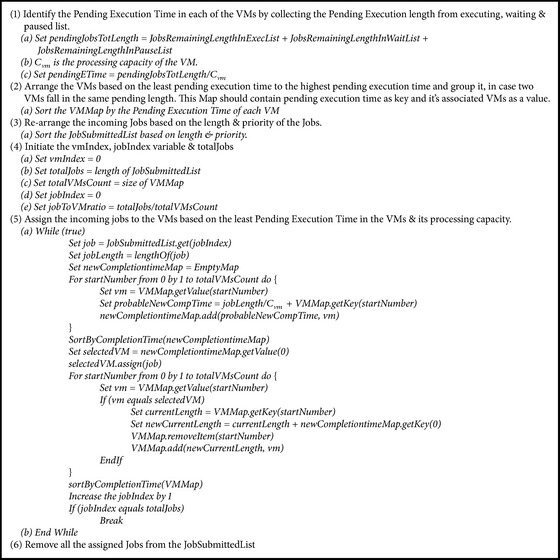
IWRR dynamic scheduler.

**Algorithm 2:**
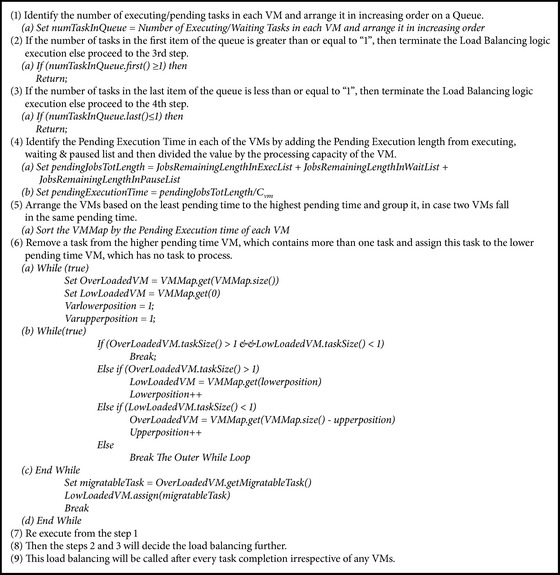
IWRR load balancer.

**Table 1:** Cloud setup configuration details.

Sl. number	Entity	Quantity	Purpose
1	Data center	1	Data center having the physical hosts in the test environment

2	Number of hosts in DC	500 hosts (200 Nos-4-CoreHyperThreadedOpteron270, 200 Nos-4-Core HyperThreadedOpteron2218 and 100 Nos 8-Core HyperThreaded XeonE5430)	Number of physical hosts used in the experiment

3	Number of process elements	8/8/16	Number of executing elements in each of the hosts. The host has 8 or 16 processing elements

4	PE processing capacity	174/247/355 MIPS	Each host has any one of the processing capacities

5	Host ram capacity	16/32 GB	Each host has any of these RAM memories

6	Number of VM	10 to 100 with an increment of 10	Number of virtual machines used in the experiment

7	Number of PE to VM	1	Processing element allotted in each VM

8	VM's PE processing capacity	150/300/90/120/93/112/105/225	Virtual machine's processing capacity

9	VM RAM capacity	1920 MB	The RAM's memory capacity of the VM

10	VM manager	Xen	The operating system runs on the physical machine to manage the VMs. It provides the virtualization

11	Number of PE in Tasks	1	The job/task's maximum usable processing element

12	Task length/instructions	500000 to 200000000	Tasks length in million instructions. The heterogeneous job length test having the variations from the mentioned minimum to maximum
